# A Rare Cause of Proptosis in Childhood: Langerhans Cell Histiocytosis

**DOI:** 10.4274/tjo.50024

**Published:** 2016-08-15

**Authors:** Mustafa Vatansever, Esra Vatansever, Erdem Dinç, Ayça Sarı, Tuba Kara

**Affiliations:** 1 Mersin University Faculty of Medicine, Department of Ophthalmology, Mersin, Turkey; 2 Mersin University Faculty of Medicine, Department of Pediatrics, Mersin, Turkey; 3 Mersin University Faculty of Medicine, Department of Pathology, Mersin, Turkey

**Keywords:** Histiocytosis, proptosis, orbital mass, childhood

## Abstract

A three-year-old male patient was admitted to the clinic with proptosis in his right eye. He had a history of fever with an unknown etiology. In examination, right proptosis was observed and an immobile mass was palpated at the lateral wall of the right orbita. Eye movement was unrestricted in all directions and anterior and posterior segment examination was normal in both eyes. On computed tomography, diffuse bone destruction and expansion was observed in the right orbital lateral wall and other cranial bones. Langerhans cell histiocytosis was diagnosed by bone biopsy. Malignancy is an important cause of proptosis in childhood. Pediatric patients who are admitted to clinic with proptosis should be carefully examined and Langerhans cell histiocytosis should also be considered as an etiology.

## INTRODUCTION

Langerhans cell histiocytosis (LCH) is the most common form of histiocytosis, with an annual incidence of 4-5 per million.^1^ The average age at diagnosis is 30.2 months and the disease occurs in males more often than females.^2^ Although both genetic and environmental factors have been implicated in its etiology, a clear relationship has not been demonstrated. Eighty percent of LCH patients have bone involvement, and half of these cases occur in the skull and facial bones. Orbital involvement occurs in 20% of LCH patients.^3^ Swelling is usually the first sign of cranial involvement due to bone expansion. The probability of organ involvement rises with lower age of disease onset; liver, lung and bone marrow involvement are associated with worse prognosis.^4^ Malignancy is etiologically important in terms of the prognosis of pediatric proptosis, and 22% of orbital lesions in pediatric patients are malignant.^5^ LCH is detected in 1-7% of orbital biopsies.^6^ Here we present a case presenting with proptosis which was diagnosed as LCH as a result of tests.

## CASE REPORT

**Figure 1:**
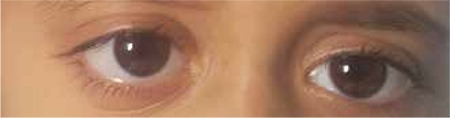
Mild proptosis of the right eye and expansion of the lateral wall of the right orbita

**Figure 2:**
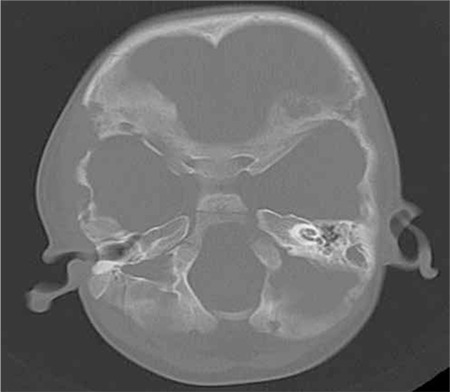
Multiple lytic lesions detected in the cranial bones on computed tomography

**Figure 3:**
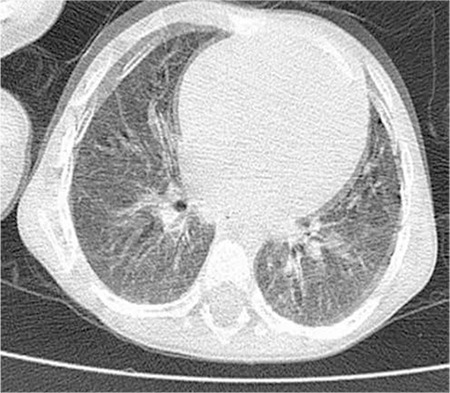
Microcystic formations visible on pulmonary computed tomography

**Figure 4:**
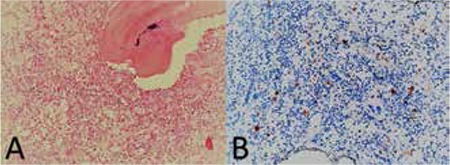
a) Characteristic Langerhans cells (hematoxylin & eosin, x200) and b) cytoplasmic CD1a staining (x200) in the patient’s bone marrow biopsy

The ophthalmology clinic was consulted regarding a 3-year-old male patient with proptosis of the right eye. It was learned that the patient had a 4-month history of persistent subfebrile temperature; tests revealed pancytopenia and hepato-splenomegaly. He was being followed in the pediatric inpatient clinic to establish an etiologic cause. On examination, the patient was able to track a light source, his eye movement was unrestricted in all directions, and anterior and posterior segment examinations were normal. Proptosis was observed in the right eye (Figure 1). An immovable mass of 1x2 cm was palpated in the lateral wall of the right orbita. Computed tomography (CT) revealed diffuse bone destruction and expansion in the right orbital lateral wall and other cranial bones (Figure 2). Microcystic formations were observed on thoracic CT (Figure 3). Suspecting histiocytosis based on the clinical and radiological findings, skeletal scintigraphy was performed. Pathologic involvement was observed in the walls of both orbita and the pelvis. The diagnosis was confirmed by pelvic bone marrow biopsy (Figure 4), which stained positive for CD1a. A treatment protocol of oral prednisolone (40 mg/m^2^/day) and intravenous vinblastin (6 mg/m^2^) was initiated. When this protocol failed, treatment was changed to a rescue protocol of cytosine arabinozid (1 g/m^2^/day) + chlorodeoxyadenosine (8.9 mg/m^2^/day). Despite some treatment response, the patient died due to pneumonia secondary to neutropenia.

## DISCUSSION

Malignant infiltrations are one of the primary diagnoses that should be considered in pediatric proptosis. Particularly in cases of proptosis accompanied by signs like leukocoria, restricted eye motility, sudden-onset asymmetric eye position, afferent pupil defect, and pseudohypopyon, malignancies should be seriously considered and must be excluded. Twenty-two percent of orbital lesions seen in pediatric patients are malignant lesions, with 65% of these being primary, 29% secondary and 6% metastatic lesions.^5^ The differential diagnosis for proptosis should include medulloepithelioma as a primary ocular tumor; rhabdomyosarcoma and optic nerve glioma as primary orbital tumors; fibroma, fibrous dysplasia and osteosarcoma as orbital bone tumors; and leukemia, lymphoma and neuroblastoma as metastatic tumors.^7^

LCH is a malignancy characterized by abnormal proliferation of Langerhans cells, but its etiology is not fully understood. Skeletal involvement occurs in 80% of LCH cases; the cranial bones are involved in more than half of these patients, while about 20% exhibit orbital involvement.^3^ The most common cranial finding is a mass located at the zygomaticofrontal suture, as in our patient. Initial ocular signs of LCH include proptosis, ptosis, eyelid edema and redness around the eyes.^8^ As in the current case, patients may present with proptosis alone. Direct imaging is the most effective way to visualize skeletal involvement in LCH, and typically reveals lytic lesions. Pulmonary involvement of LCH begins as nodular lesions that develop with disease progression into thin-walled cystic structures which are easily detected by high-resolution CT.^9^ Biopsy of these nodular lesions is recommended for histopathologic confirmation of pulmonary LCH. However, the Histiocytosis Society reported that the observation of typical cystic lesions on high-resolution CT is sufficient to diagnose pulmonary involvement.^10^ Our patient presented with typical lytic bone lesions as well as microcystic pulmonary lesions, so lung biopsy was deemed unnecessary. The Histiocytosis Society also created a guideline for diagnosing splenic and hepatic involvement through clinical examination and ultrasonography, without histopathologic sampling.

LCH most often arises in childhood but onset is known to occur in patients of every age group. Prognosis worsens with lower age at diagnosis. Clinical manifestation varies from benign unifocal skeletal involvement to aggressively malignant multisystem disease. Prognosis is poor in cases with disease spread and organ dysfunction.^11^ For patients with unifocal skeletal involvement, local treatment or observation may be adequate.^8,12^ However, these patients should be followed closely due to the risk of progression to multisystem disease. Systemic chemotherapeutic agents are used to treat multisystem LCH. The current case exhibited multifocal skeletal involvement; despite the absence of hematopoietic, hepatic, splenic, or pulmonary involvement, he was considered at risk for multisystem disease and systemic chemotherapy was initiated. Patients with hematopoietic, hepatic, splenic or pulmonary involvement are considered at risk for multisystem disease in the risk stratification system of the Histiocytosis Society, and treatment protocols were designed accordingly.^10^ The prognosis of LCH patients with multisystem involvement is poor.

Due to the inability of pediatric patients to express themselves and the subtlety of the signs and symptoms of orbital malignancy, the families of these patients may overlook or disregard their symptoms. This may lead to delayed diagnosis and worse prognosis. Unfortunately, our patient’s family disregarded his proptosis and orbital mass.

## CONCLUSION

All pediatric patients presenting with proptosis should undergo a thorough examination, and the possibility of an underlying malignancy should be considered even in cases with subtle signs. It should be kept in mind that LCH is among these malignancies and that an initial diagnosis is possible with careful radiologic imaging.

### Ethics

Informed Consent: It was taken.

Peer-review: Externally and internally peer-reviewed.
